# Interface Between Newly Formed Dentine and Mineral Trioxide Aggregate: A Preliminary Scanning Electron Microscopy Study

**Published:** 2006-04-01

**Authors:** Masoud Parirokh, Saeed Asgary, Mohammad Jatar Eghbal, Sally Stowe, Jamileh Ghoddusi

**Affiliations:** 1 *Department of Endodontic, School of Dentistry, Kerman University of Medical Sciences, Kerman, Iran*; 2 *Department of Endodontics, Dental Research Center, School of Dentistry, Shahid Beheshti Medical University, Tehran, Iran*; 3 *Facility Coordinator, Electron Microscope Unit, Research School of Biological Sciences, Australia National University, ACT, Australia*; 4 *Department of Endodontic, School of Dentistry, Mashhad University of Medical Science, Mashhad, Iran*

**Keywords:** Dentinogenesis, MTA, Pulp Capping, SEM

## Abstract

**INTRODUCTION:** This study was carried out to investigate calcific tissue formation against mineral trioxide aggregate (MTA) after pulp capping.

**MATERIALS AND METHODS:** The pulps of six teeth from four dogs were exposed and capped with MTA. After extraction each tooth was sectioned into halves. Each half was then further sectioned in the mesiodistal or buccolingual direction. Calcified tissue of the capped area was examined from either the pulpal side or in profile view using scanning electron microscopy and focused ion beam imaging.

**RESULTS:** The results from all views showed that MTA crystals were in direct contact with calcified tissue. Calcified bridges after focused ion beam preparation showed the same pattern that were seen by scanning electron microscopy in profile view. Newly deposited dentine and early phase of calcosphirit structures could clearly be distinguished from older tubular dentine.

**CONCLUSION:** MTA when used as a pulp capping agent could produce calcific tissue in underlying pulp.

## INTRODUCTION

Mineral trioxide aggregate (MTA) has been introduced as a material of choice for pulp capping, perforation repair, apexification and root-end-filling (1-9). Studies show that MTA exhibits minimal cytotoxicity, excellent marginal adaptation and high biocompatibility (10,11).

Although freshly mixed MTA has more toxic effect than its set form (12), there is no difference in the quantity of cementum or osseous healing associated with freshly placed or set MTA when used as a root-end filling material (13). Research has indicated that MTA could be considered cemento conductive (14) and dystrophic calcification may be present in connective tissue adjacent to MTA (15).

In several animal research studies MTA has demonstrated more beneficial characteristics as a direct pulp-capping agent than calcium hydroxide {Ca (OH)2} controls (3-5,16). The studies showed complete calcified bridge formation with a mild inflammatory reaction in the majority of samples treated with MTA. Ln comparison with Ca (OH) 2, MTA can produce significantly more dentinal bridging in a shorter period of time with less associated inflammation. Most recent studies on the direct pulp capping effect of MTA were histological and therefore focused on inflammatory reaction of pulp and dentinal bridge formation. Scanning electron microscope (SEM) and X-ray analysis of the capping area were used (6,17).

Focused ion beam (FIB) system is similar to SEM, except that instead of an electron beam, a beam of ions is scanned across the sample. The ion beam interacts strongly with the sample, producing local etching and deposition of material. In a gallium ion system the ion beam is ejected from a liquid metal source, with a spot size that can be as low as 10 mm on modern systems. The ion beam allows precise milling at well-localized sites. This allows obtaining cross­sectional images of small structures (19).

The purpose of this study was to examine the interface of MTA and the newly formed dentinal bridge using SEM and FIB.

## MATERIALS AND METHODS

Six teeth from four healthy 18-24 months old Beagle dogs were used in this study. Under general anesthesia with an intramuscular injection of 20 m g/kg Ketamine HCI (Alfasan, Woerden, Holland) and 0.2 mg/kg Xylazine (Bayer, Munchen, Germany), the teeth were rinsed with 0.2% chlorhexidine. After local anesthesia through an infiltration injection containing mepivacaine 3% (SPEE, Germany), each tooth was isolated with a rubber dam. Using a No.1 round bur in a high speed hand piece and copious water spray, cavities were prepared in the labial surface of the teeth and standardized pulp exposures (1 mm in diameter) were created.

Bleeding was controlled using sterile saline and cotton pellets before placing MTA as the pulp capping material. MTA (ProRoot MTA Dentsply, Tulsa Dental, Tulsa, OK, USA) was mixed according to the manufacturer’s directions. Then the exposure sites and the cavity were covered and filled with MTA.

After two weeks, vital perfusion fixation was performed using Karnovsky solution. The teeth and their surrounding tissues were then removed. For further fixation after splitting the roots with a mallet and chisel they were placed in 2.5% glutaraldehyde solution for fourteen days. Each tooth was sectioned in half. Each half was then further sectioned in the mesiodistal (group A) or buccolingual direction (group B). All samples were then immersed in 5.25% sodium hypochlorite for one hour.

For SEM observations the samples were gold coated. A rectangular area at the border of the capping area in group A teeth was etched with the FIB for up to 15 hours at a current of c.350pA. All samples in both groups were observed with a Field Emission SEM (Hitachi 4500, Hitachi, Japan) and scanning electron Microscope (JEOL 6400, JEOL, Japan). The images were digitally recorded from slow scan "photographic" output as 1024 x 768 pixel images using an Image Slave SEM image capture system (OED Scientific, Sydney, Australia).

## RESULTS

Newly formed hard tissue structures were found in all but one sample after two weeks of pulp capping with MTA. SEM images of the samples viewed from the pulpal side showed that the calcific tissue in the capping area was different from the original dentine.

The surface of the calcific area was extremely rough with many projections and crevasses. Therefore newly deposited dentine and early phase of calcospherite structures could clearly be distinguished from older tubular dentine.

**Figure 1:**
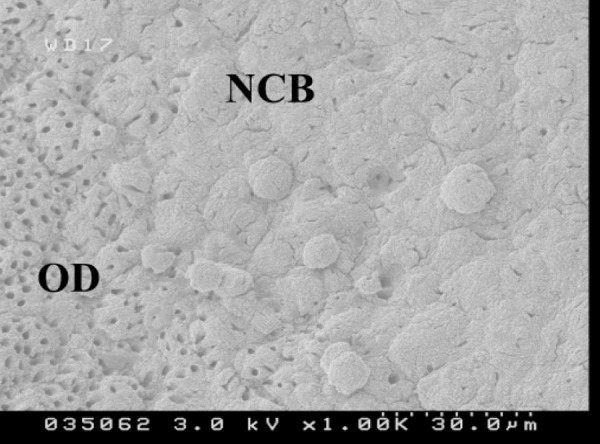
Old dentin (OD) showing its highly tubular appearance in comparison with recently formed calcified bridge (CB). (Magnification 1000×-Hitachi 4500 FESEM)

Although early phase of calcospherites structures contain many tubular structures, the number of tubules are less than mature older dentin. Between the less tubular central area of the bridge and the tubular surrounding area was an intermediate zone of less tubular dentin (Figure 1). Many cracks were observed in the calcific and sound dentine of the capping area.

**Figure 2:**
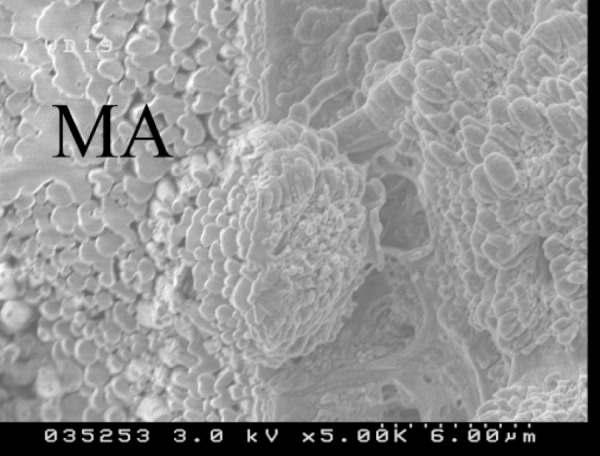
FIB milling in the capping area showing columns of different sizes. Milling area showed melting dentin (MD). (Magnification 5000×-Hitachi 4500 FESEM)

Although some beam damage was evident, when the superficial layer of the dentine was removed by the FIB, the columnar structure with different sizes of columns could be recognized (Figure 2). In the bridge area, FIB revealed globular deposition of dentine under a superficial layer.

**Figure 3:**
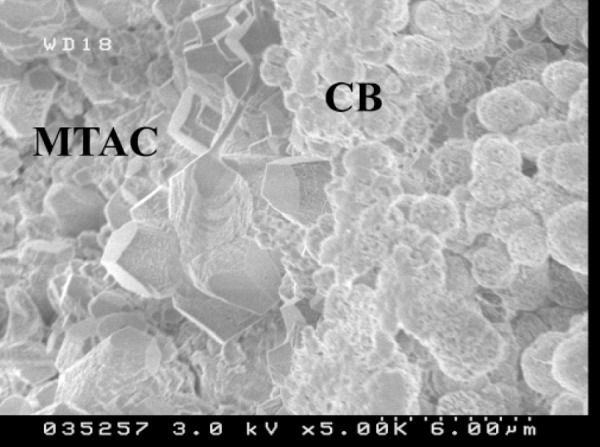
Profile view of capping area showing MTA crystals (MTA) in direct contact with calcified bridge (CB). (Magnification 5000-Hitachi 4500 FESEM)

Specimens cut through the buccolingual direction (that is in "profile" view) and viewed with SEM also showed the globular structure of the calcified bridge. Direct contact of MTA crystals with calcified tissues could be observed (Figure 3).

## DISCUSSION

Various materials have been used for pulp capping. Among them, the most promising is MTA as experimentally demonstrated in several animal studies (3-5).

The results of this study showed that MTA was in direct contact with the calcified bridge. Previous researches have shown the biocompatibility of MTA, with cementum deposition directly apposing it (20,21). Direct contact of hard tissue with MTA (Figure3) showed that the calcified bridge could form on MTA without the presence of a necrotic zone, debris and other sort of materials between them.

In this study SEM images showed non-tubular structures in the middle of the calcified bridge after two weeks along with some tubular structures particularly at the border of the capped area. The profile view of the capped area showed that calcified tissue contact with MTA crystals has a globular pattern. Although the SEM images from the pulpal side of the capped area could not show the same structure, SEM images after using FIB showed a similar pattern.

Previous studies have shown that pulp responds favorably to protection by a MTA layer (3-6,16). The reparative dentin under MTA was consistently thicker and more uniform compared with calcium hydroxide (3,4).

The presence of bacteria and their by-products is one of the most important reasons for failure of pulp capping (3). Cox and associates have shown that pulp healing is more dependent on the capacity of pulp capping material to prevent microleakage rather than the specific properties of the material itself (22). Previous comparative studies on MTA and calcium hydroxide have emphasized a significantly higher frequency of calcified bridge formation and less inflammation in MTA specimens (3-6,23). Interesting result of present study in production of calcific bridge after a short period of pulp capping may be due to use of MTA as a dressing material. Previous research studies have also shown that MTA has excellent sealing ability (1,2).

Sarkar and associate in a recently published study have shown that endodontically prepared teeth, filled with mineral trioxide aggregate and stored in synthetic tissue fluid, produced at the dentin wall an adherent interfacial layer compositionally similar to hydroxyapatite. They conclude that MTA is not an inert material in a simulated oral environment; it is bioactive. In contact with synthetic tissue fluid, it dissolves, releasing all of its major cationic components and triggering the precipitation of hydroxyapatite on its surface and the surrounding fluid.

It appears to bond chemically to dentin when placed against it, possibly via diffusion-controlled reaction between its apatitic surface and dentin. They believe that the clinical success of MTA, in terms of its seal ability, biocompatibility, and dentinogenic activity is rooted in the aforementioned physicochemical reactions (24).

Major (25) states that the presence of a calcified bridge may not be a suitable criterion for assessment of successful pulp healing. Previous research on calcium hydroxide as a pulp capping material has shown that the calcified bridge at the pulp capping site is imperfect and cannot provide a hermetic seal that would protect the pulp against bacterial microleakage (26). The imperfections, called tunnel defects, involve multiple perforations that allow communication between the pulp surface and capping material interfaces. In this study tunnel defects could not be seen in samples in either profile or pulpal view. However, the calcified bridge, when etched slightly with FIB, showed spaces between the globular deposits of the hard tissue. The gaps are expected to fill in over time.

In this study many cracks could be seen in the calcific area. The previous SEM studies on teeth have shown that the cracks might be formed during SEM preparation (17,18). It has been recommended to use environmental SEM to overcome artifacts during SEM studies (27).

In conclusion this study showed early events after pulp capping with MTA, further studies with longer durations are needed to support these findings.
